# Changes in climate patterns and their association to natural hazard distribution in South Tyrol (Eastern Italian Alps)

**DOI:** 10.1038/s41598-020-61615-w

**Published:** 2020-03-19

**Authors:** Romy Schlögel, Christian Kofler, Stefano Luigi Gariano, Jean Van Campenhout, Stephen Plummer

**Affiliations:** 1 ESA Climate Office, ECSAT, Didcot-Harwell, OX11 0FD United Kingdom; 2 Institute for Earth Observation, Eurac Research, Bozen, 39100 Italy; 3 CNR IRPI, Perugia, 06127 Italy; 4 Department of Geography, University of Liege, Liege, 4000 Belgium; 5 Faculty of Science and Technology, Free University of Bozen-Bolzano, Bozen, 39100 Italy; 6 Present Address: Division for Satellite Analysis and Applied Research (UNOSAT), UNITAR, United Nations Office at Nairobi, Nairobi, 00200 Kenya; 7 Data Applications Division, ESRIN, Frascati, 00044 Italy

**Keywords:** Climate sciences, Natural hazards

## Abstract

In Alpine regions changes in seasonal climatic parameters, such as temperature, rainfall, and snow amount have already been observed. Specifically, in the South Tyrol area, meteorological observations indicate that temperatures are increasing and the number of snow days has generally diminished over time with perennial snow line now observed at higher elevations. Changes in rainfall have also been observed with more events associated with higher temperatures in the summer season. Natural hazards - mainly debris and mud flows, landslides, avalanches, rock falls, and (flash) floods - that affect this area every year, damaging population and infrastructures, are either weather or cryosphere-related. While these events have been recorded sporadically since the beginning of the 20th century, a systematic approach of their inventory has been done by local authorities since the 1990s. So far, Earth observation data has not been exploited to complete or complement existing inventories nor have they been used to investigate the influence of climate perturbation on potentially dangerous natural phenomena. The research presented here thus has three objectives: (i) analyse long time series of climate data and hazard occurrence in South Tyrol to examine if these records exhibit a coherent response of hazards to changes in climate; (ii) measure the spatio-temporal evolution of climatic and natural hazard events recorded, and (iii) explore potential relations between meteorological conditions and the hazard occurrence. In this context, in-situ and satellite-based climate data are exploited to study natural hazard triggers while the potential of Earth observation data is evaluated as a complement to the existing historical records of natural hazards. Specifically, Copernicus Sentinel-1 images are used to detect the spatio-temporal distribution of slow earth surface deformations and the results used for checking the completeness of the actual slow-moving landslide inventories. Hazard-related changes in the South Tyrolian landscape have also been analysed in relation to particular meteorological events at a regional scale, assessing trends and anomalies. Results show that: (i) satellite data are very useful to complement the existing natural hazard inventories; (ii) in-situ and satellite-based climate records show similar patterns but differ due to regional versus local variability; (iii) even in a data-rich region such as the analysed area, the overall response of natural hazard occurrence, magnitude, and frequency to change in climate variables is difficult to decipher due to the presence of multiple triggers and locally driven ground responses. However, an increase in the average annual duration of rainfall events and debris flow occurrence can be observed.

## Introduction

Mountains have been acknowledged as “sentinels of environmental change” because they have physical dynamics that are readily identifiable and respond more rapidly than other geographical entities to environmental change^1^. In several areas of the world they have been indicated to be highly susceptible to the impacts of a changing climate. According to the Intergovernmental Panel on Climate Change^2^, there is a “high confidence that changes in temperature, glacial retreat, and/or permafrost degradation will affect slope instabilities in high mountains, and medium confidence that temperature-related changes will influence bedrock stability”. Furthermore, there is “medium confidence that high mountain debris flows will begin earlier in the year because of earlier snow-melt, and that continued mountain permafrost degradation and glacier retreat^3^ will further decrease the stability of rock slopes”. In high mountain areas, not only small-sized rock falls and ice falls, but also large rock slides and rock avalanches may become more abundant^4,5^. Higher levels of precipitation raise the potential for higher pore-water pressures in unstable volumes of rock and debris, e.g. promoting landslide formation^6^. Other research^7,8^ has demonstrated a link between slope failures, preceding, anomalously warm episodes in mountainous areas, and rapid thaw processes.

The study of the impacts of climate change on gravitational hazards can be attempted by adopting modelling, empirical, or the combination of both methods^9^. Regarding the empirical approach, a number of investigators have analysed historical records of event occurrence, and compared them to meteorological and climatic variables, mostly rainfall and temperature^10–12^. The majority of this work has focused on debris flows, shallow landslides and rock falls that occurred in areas characterised by a high mountain and mountain morphological setting, covering different periods in the range from mid-19th century to the present. However, while there is a theoretical understanding that gravitational hazards respond to changing climate conditions, it is still difficult to detect changes over mountainous regions in the observational records^13,14^, and often the results are contradictory or uncertain^9^. Some researchers^5,15–17^ have attempted to analyse the relationship between climatic variables and gravitational hazards (mostly, rock falls and debris flows) in the Alps, particularly in the Italian Alps, mentioning limitations due to complex mountainous systems and incompleteness of records.

Over the last two decades, Earth Observation (EO) has become a common technique for environmental data collection for mapping hazards and disasters^18^. Klemes^19^ already stated that EO is a promising technique to investigate the extreme variability of hydrological and meteorological variables in mountainous terrain. Both Optical and Synthetic Aperture Radar (SAR) data have been used to map natural hazard events but, even to date, most mapping operations still rely on manual and time-consuming interpretation techniques to achieve a high accuracy of process understanding^20^. SAR data have the advantage (compared to optical imagery) to be, in principle, unaffected by cloud cover, which is often present in mountainous areas. Differential SAR interferometry (DInSAR) is particularly useful for the detection and monitoring of slow ground deformations^21^.

New constellations of short revisit time satellites (e.g. Copernicus Sentinels^22^) are beginning to provide sufficient information to locate and monitor ground surface changes^23^ and deformations as well as rates of the processes. Tools to process the huge data quantity offered by the Copernicus Sentinels for hazard assessment (e.g.^24^) are already available and are being continuously developed in terms of processing methodology and sensor availability. In addition EO measurements of value for the study of climate and climate change have started to become available for example through the European Space Agency’s Climate Change Initiative (CCI)^25,26^. These datasets are generally relevant for global use but in some cases can be applied to a complex mountainous context^27–29^.

**Figure 1:**
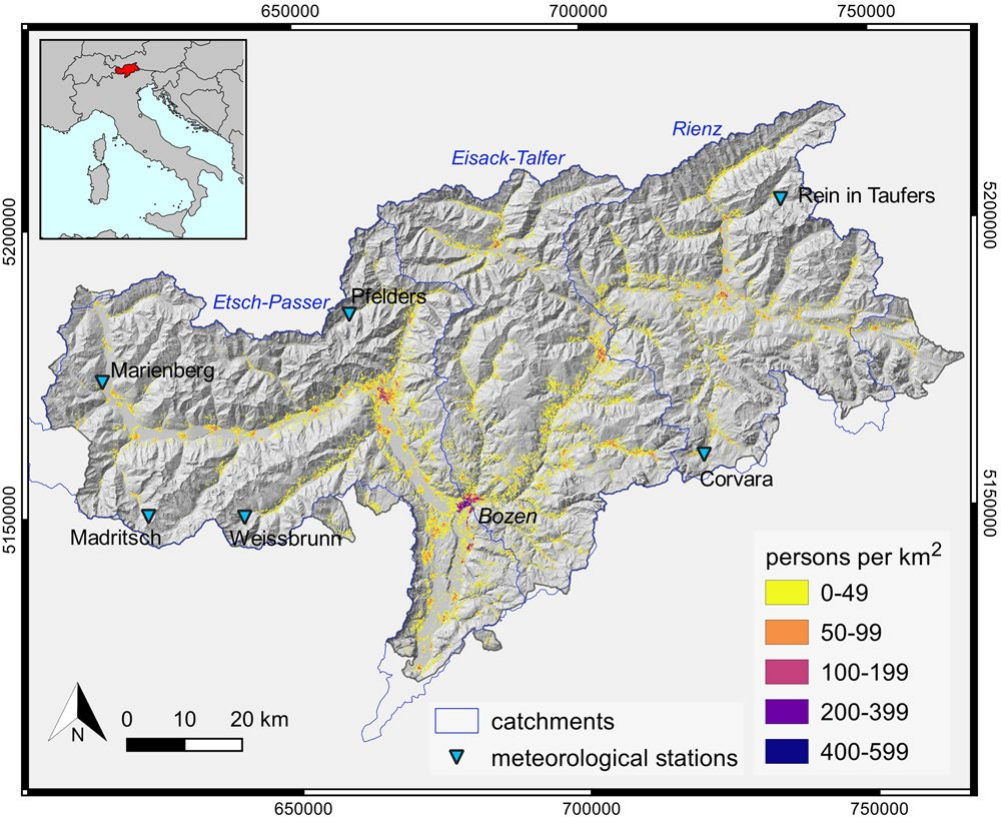
Location of South Tyrol (Autonomous Province of Bozen/Bolzano). The meteorological stations used, the main catchments and the mean resident population of the year 2015 ($1/k{m}^{2}$) are shown; the population was modelled from data provided by the Department for Monitoring of the Labour Market of the provincial government^33^. Map realised with QGIS Geographic Information System, Open Source Geospatial Foundation Project (http://qgis.osgeo.org). Background digital elevation model downloaded from the WebGIS service of the South Tyrolean public administration (http://geokatalog.buergernetz.bz.it/geokatalog/). Data available under the Open Database License (© OpenStreetMap contributors); cartography licensed as CC BY-SA (https://www.openstreetmap.org/copyright/en).

The aim of this paper is to try to understand if changes in climate in complex mountainous areas can be observed to have an impact on the distribution, frequency and magnitude of natural hazards. The area of study is South Tyrol (Autonomous Province of Bozen/Bolzano), Northern Italy (Fig. 1). This populated alpine region is strongly affected by natural hazards (NH): every year several weather-related NH such as debris flows, landslides or avalanches, are recorded^30–32^. In particular, this research aims to provide insights in answering several questions. Is South Tyrol showing evidence of a changing climate in terms of extremes and trends which could be associated with similar changes in NH according to a consistent spatial pattern? Are ground-based records sufficient to obtain answers in the mountainous environment? What complementary information can be provided from EO if ground-based data are insufficient? This last objective is important because it allows the transfer of monitoring capability to other mountainous regions with only sparse in situ information.

This study is structured to allow the assessment of similarities and differences between various sources of information on NH in terms of observed climate trends and weather anomalies. Although existing inventories of NH events are known to be mostly incomplete, they give valuable insights on impacted areas and the changing occurrence of NH over time. As part of this analysis, SAR techniques are also assessed to examine how they could contribute to completing slow instability inventories especially in high elevations, where the existing inventories are limited. Large events in these high elevation areas could potentially have dramatic impacts downstream in response to, for example, in case of rapid snow melting possibly combined with heavy rainfall. A concerted effort is therefore needed to bring together (i) historical records and remote sensing data of NH occurrence/activity, and (ii) ground/in-situ measurements and satellite observations of meteorological variables to examine whether insight can be obtained on the interaction between climate and NH. A number of climate-related parameters play a significant role in NH triggering. Among them, rainfall is used in most of the analysis here as it constitutes the longest and most consistent record of the available time series.

In order to study their single and combined influences the following tasks are addressed in the subsequent sections: (i) the spatial and temporal features of recorded NH (e.g. density, frequency, trend) are evaluated; (ii) the characteristics of rainfall events reconstructed from ground-measured long time series are investigated; (iii) the return periods of extreme rainfall events, recorded from various sources at different locations, are calculated and analysed; (iv) the presence/absence of trends of weather-related variables, changing with location, is investigated, and their statistical significance is assessed; (v) the seasonal and annual climate variable anomalies are evaluated; (vi) maps showing different types of hazard, their density and slope deformation hot-spots with Earth Observation and existing catalogues are prepared and analysed (vii) finally, spatial and temporal relationships between weather-related phenomena and NH occurrence are assessed and discussed.

## Results

### Weather and climate variations

#### Rainfall analysis

**Figure 2:**
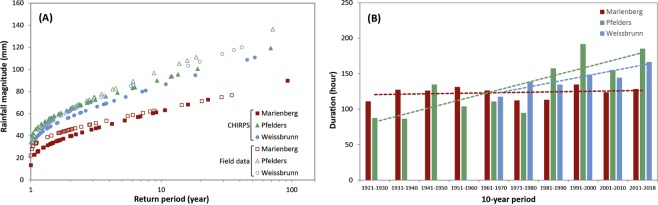
Return period and trend of extreme rainfall events. (**A**) Comparison of return periods of rainfall magnitudes over satellite and field data recorded at the different stations. (**B**) Comparison of average duration (in hour) trends (in hour) over 10-year periods for the different stations.

A first analysis compares Gumbel curves (Fig. 2A) for both satellite (e.g. Climate Hazards group Infrared Precipitation with Stations, CHIRPS) and field measurements at the stations of Marienberg (North-West; 1323 m a.s.l), Pfelders (North; 1623 m a.s.l) and Weissbrunn (South-West; 2297 m a.s.l) (Fig. 1). The Gumbel curves show that the magnitudes of rainfall events in field measurements are different from one station to another. For instance, 10-year rainfall is estimated at 91 mm with CHIRPS data and 99 mm with ground-based data in Pfelders whereas Weissbrunn station gives values of 85 mm and 95 mm, respectively and in comparison, Marienberg shows the lowest values of 62 and 64 mm (with CHIRPS and ground-based data, respectively).

While CHIRPS satellite data for the same sites generally underestimate ground-based observations, because of their resolution, they slightly overestimate low magnitude events registered at Pfelders and Weissbrunn and record the same trend in the Gumbel curves (Fig. 2A). Rainfall events with a 40 mm-threshold are recorded every 1–2 years for each station whereas those of 60 mm are measured every 1.65 (1.7) years (Pfelders), 1.8 (2.3) years (Weissbrunn) and 6.8 (8.4) years (Marienberg), as recorded in field and satellite (parentheses) data. Considering only ground-based data, disparities between stations are even higher for 70 mm rainfall events with a return period of 2.5 to 2.8 years at Pfelders and Weissbrunn stations, respectively while Marienberg exhibits a return period of 17.8 years.

A further analysis of the field measurements considers 2290, 2897, and 1687 rainfall events identified for Pfelders (1921–2018), Marienberg (1924-2018), and Weissbrunn (1961–2018) stations, respectively (Fig. 2B). The distribution of the rainfall events is similar for the three sites, with the highest number of events between June and August. Considering the entire periods for the three stations, the average values for the duration and the cumulative rainfall of the events are shorter/lower at Marienberg (124 h, 22.5 mm), medium at Pfelders (133 h, 36.7 mm) and longer at Weissbrunn (143 h, 35.8 mm). To investigate possible changes in the distribution of the rainfall events in the analysed period, the reconstructed rainfall events were grouped in periods with different lengths: 30-year, 20-year, 10-year, 5-year periods. The statistical significance of any trends has been assessed using the Mann-Kendall non-parametric test^34,35^(Table 1). Considering the annual number of events, a slightly decreasing trend (0.05 level of significance) is found for Pfelders and Weissbrunn ($-0.1$ event/year) and no trend is found for Marienberg. The average annual duration of rainfall events strongly increased (increasing trend with 0.001 level of significance) at Pfelders (+1.1 hours/year) and Weissbrunn (+0.9 hours/year), while it remained stable in Marienberg (slope of the linear regression curve = 0.066). The average annual value of cumulative rainfall increased at Weissbrunn (increasing trend of 0.5 mm/year with 0.001 level of significance) and remained stable at the other two sites. These variations are visible in all temporal groupings; however, they are the clearest in the 5-year analysis.

**Table 1:** Upward (+) and downward ($-$) trends for annual number, duration, and cumulative rainfall of reconstructed rainfall events, in the reported periods. Values in the brackets are the levels of statistical significance of the trends.

	Weissbrunn	Pfelders	Marienberg
Rainfall events	1961–2018	1921–2018	1924–2018
Annual number	$-$ (0.05)	$-$ (0.05)	no trend
Duration	+0.001)	+(0.001)	no trend
Cumulated rainfall	+(0.001)	no trend	no trend

These event-based rainfall analyses were not carried out for Corvara, Madritsch and Rein in Taufers stations, given that they have short time series (see Supplementary Material) also with some missing data.

#### Changing climate variables

**Figure 3:**
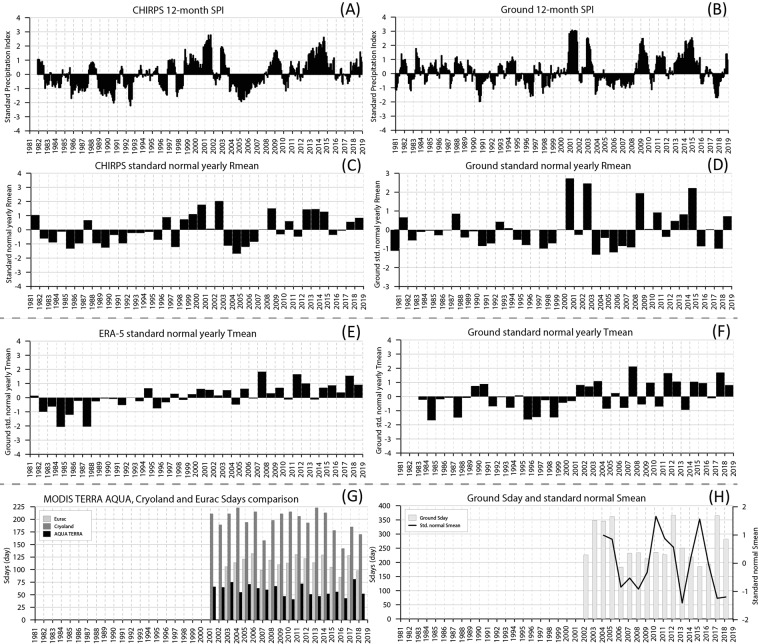
Comparison of satellite (**A,C,E,G**) and field (**B,D,F,H**) climate variables for Weissbrunn. Evolution from 1981 to 2018 of 12-month SPI (**A,B**); yearly average rainfall standard mean (**C,D**); standard mean yearly average temperature (**E,F**); winter seasons snow days (Sdays) (**G**) and yearly snow height standard mean (**H**).

The evolution of the Standardized Precipitation Index over 12 months (SPI-12) and standardised means of rain, temperature and snow over time is shown for the Weissbrunn station in Fig. 3 for both satellite-derived (CHIRPS) and ground-based observations. The parameters extracted at other stations (located in Fig. 1) are presented in the additional material. Weissbrunn is shown here because it is the highest station with the longest time series. The other sites are not always similar to the Weissbrunn station. For example, very wet and extremely wet years are observed in 2001, 2009 and 2014 years using SPI-12 at Marienberg and Weissbrunn (with a good match between ground and re-analysed data; see Fig. 3A,B) but at Pfelders, only the 1982 and 2001 years are clearly visible on CHIRPS rainfall estimates data. According to all datasets, the 2004-2008 period can be considered as dry whereas the period from 2012 to 2014 records a large number of wet events. However, the CHIRPS standard normal yearly average rainfall standard mean (Rmean) shows low but constant values during the 2003-2006 period (Fig. 3C), which contrasts with ground measurements that exhibit an extremely dry Rmean in 2003, consistent with the 2003 drought in Europe. The minimum, mean, and average values of SPI-12 show a statistically significant increasing trend (0.01 level of significance, assessed using the Mann-Kendall test) for all values in Weissbrunn, while no significant trend is found for Pfelders (Table 2).

**Table 2:** Upward (+) and downward ($-$) trends for minimum, mean, and maximum values of SPI-12, calculated in the period 1982–2018. Values in the brackets are the levels of statistical significance of the trends.

SPI annual value	Weissbrunn	Pfelders	Marienberg	Madritsch	Corvara	Rein in Taufers
minimum	+(0.01)	+(0.05)	+(0.1)	+(>0.1)	$-$(>0.1)	$-$(>0.1)
mean	+(0.01)	+(0.05)	+(0.05)	+(>0.1)	$-$(>0.1)	$-$(>0.1)
maximum	+(0.05)	+(>0.1)	+(0.05)	+(>0.1)	$-$(>0.1)	$-$(>0.1)

ERA-5 mean (e.g. Fig. 3E), and maximal daily temperatures represent a great alternative for monitoring yearly and seasonal variability and anomalies because they do not have gaps in data that characterise individual ground measurements. ERA-5 data show indisputable increases of mean and maximum temperatures over time, especially for spring months (see Supplementary Material). Peaks of low yearly means were observed in 1980, 1984 and 1987 (Marienberg and Weissbrunn), in 1995-1997 and lately in 2009-2010 (Pfelders), but yearly standard Tmeans are all positive since 2011 (Fig. 3E). The mean annual temperatures, gathered from both ERA-5 and ground data, show increasing trends for all stations (Table 3). Most of the trends are characterized by high levels of statistical significance according to the Mann-Kendall test.

**Table 3:** Trends for the mean temperature variable, both from in-situ measurements on remote estimates (${}^{\circ }$C/year). Values in the brackets are the levels of statistical significance of the trends, according to the Mann-Kendall test; the lower the value is, the higher the significance of the trend.

Annual Tmean	Weissbrunn	Pfelders	Madritsch	Corvara	Rein in Taufers
ERA-5	+0.07 (0.001)	+0.07 (0.001)	+0.07 (0.001)	+0.08 (0.001)	+0.07 (0.001)
Ground	+0.02 (>0.1)	+0.0 (0.01)	+0.02 (0.05)	+0.04 (0.05)	+0.09 (>0.01)

Due to the extensive cloud coverage no matter the season considered, MODIS satellite data are difficult to use for monitoring spatial variability over time, even considering interpolation in the Cryoland products between snow falls (see climate data in Supplementary Material). Moreover, the data are only available for a limited period (>2001) which hampers temporal analysis of snow cover fraction (FSC) (Fig. 3G). Nevertheless while the yearly snow height standard mean (Smean) does not show any apparent decreasing trend (see additional material), over this period, at the Pfelders station, the number of winter season snow days (Sdays) have diminished, especially the last 4 years.

### Multi-temporal analysis of slope deformation based on inventory and earth observation data

#### Spatial variability

**Figure 4:**
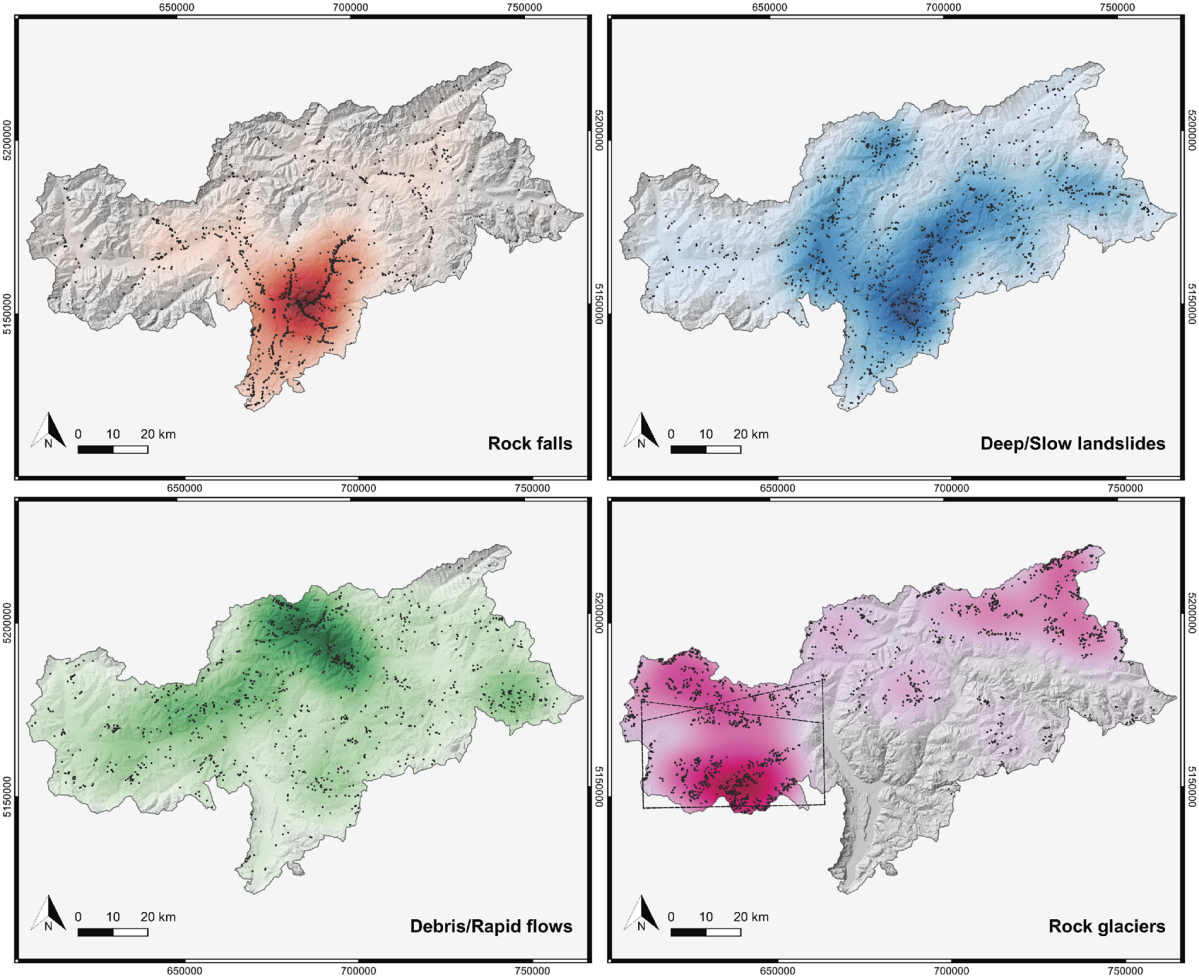
Density maps (heatmaps) of various slope instability processes over the South Tyrol province. Coloured areas represent the sites with higher density of slope instability processes in each category (black dots). Maps realised with QGIS Geographic Information System, Open Source Geospatial Foundation Project (http://qgis.osgeo.org). Background digital elevation model downloaded from the WebGIS service of the South Tyrolean public administration (http://geokatalog.buergernetz.bz.it/geokatalog/). Data available under the Open Database Licence (© OpenStreetMap contributors); cartography licensed as CC BY-SA (https://www.openstreetmap.org/copyright/en). The dotted line box shows the SAR (ascending and descending passes) mapping extent analysed in Fig. 6.

**Figure 5:**
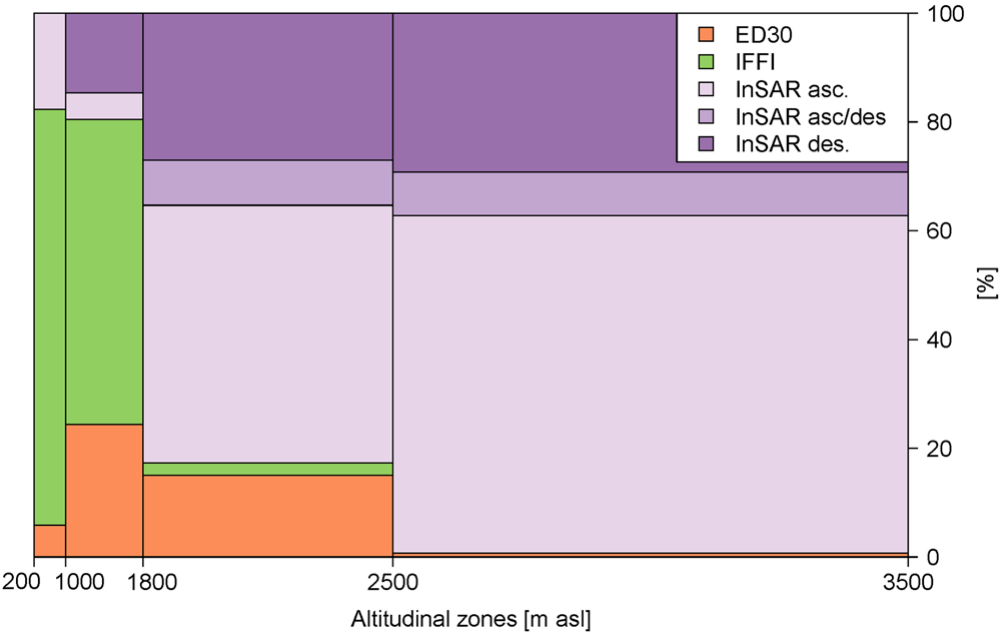
Distribution of events over the altitude and relative frequencies of recordings from IFFI and ED30 catalogues and MTA for the years 2017-2018.

The two available inventories for NH events, namely *Inventario dei Fenomeni Franosi in Italia* (IFFI) and *EreignisDokumentation 30* (ED30), were merged and investigated spatio-temporally with recent land surface processes detected with multi-temporal InSAR analysis (MTA) to investigate their complementarity. Figure 4 - generated based on IFFI and ED30 - shows the spatial distribution of various hazard types over South Tyrol and it is clear that the distributions of each NH type are very different. Specifically, rock fall is more present in the southern part while a high density of landslides can be identified in the eastern and northern part of the province. Debris flows are mainly observed in the North and the West. In addition, although not necessarily a hazard, rock glaciers show a high density in the western part of South Tyrol (information on the spatial distribution of rock glaciers was extracted from the inventory created by Bollmann *et al*.^36^). Locations of the various events were analysed according to elevation (see Fig. 5). This shows that events recorded in the databases are mostly located at lower altitudes (i.e. up to 1800 m a.s.l. about 80% of the records in the period 2017–2018 come from IFFI or ED30), while at higher altitudes, observations (excluding South and North facing slopes with angles above 40 degrees) made with MTA are prevalent.

**Figure 6:**
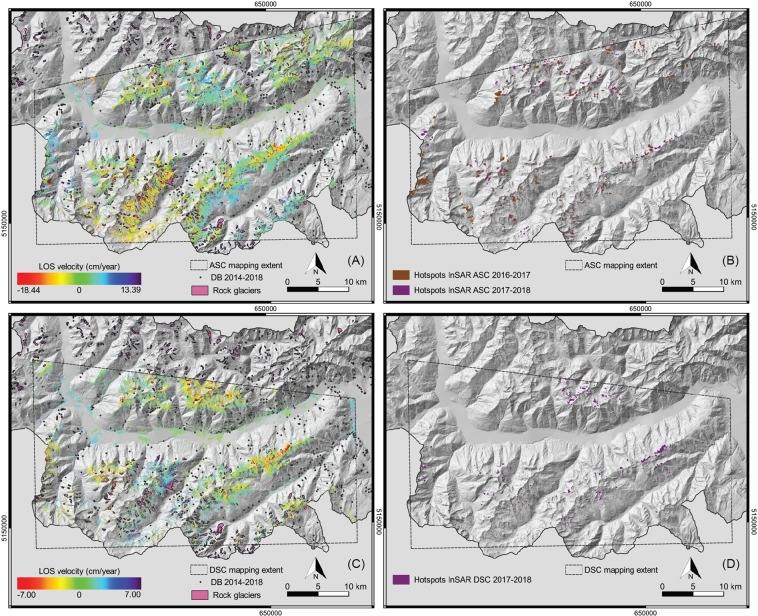
Deformation maps (**A,C**) and active hotspot areas (**B,D**) mapped from Sentinel-1 images processed with the FASTVEL MTA algorithm available in the Geohazard Exploitation Platform (GEP) compared to rock glaciers and natural hazard events recorded. Maps realised with QGIS Geographic Information System, Open Source Geospatial Foundation Project (http://qgis.osgeo.org). Background digital elevation model downloaded from the WebGIS service of the South Tyrolean public administration (http://geokatalog.buergernetz.bz.it/geokatalog/). Data available under the Open Database Licence (© OpenStreetMap contributors); cartography licensed as CC BY-SA (https://www.openstreetmap.org/copyright/en).

In SAR ascending mode, the MTA maps 325 active areas (hotspots) during the period 2017–2018 (Fig. 6) while 224 areas were mapped for the period 2016–2017. According to the IFFI database, 220 mass movements were recorded during the period 2014–2018 for the same area, with 47, 20 and only 8 events in 2016, 2017 and 2018 respectively. In descending mode, 218 hotspots areas were mapped during the period 2017–2018 including 54 areas partially overlapping the ascending inventory according to the topography and the different SAR geometries. However, not enough images were available to complete the descending MTA successfully for the period 2016–2017.

Considering both tracks, more hotspot areas (i.e. areas moving of more than 2 cm per year) were observed with MTA than in the IFFI database which means that this catalogue definitely shows a lack of information on slow moving processes. In the 325 records during the 2017–2018 period (in ascending mode), only 5 events may correspond to IFFI events, possibly 6 to some from the ED30 and 60 to some (active) rock glaciers. In addition, between 65 and 87 rock glaciers have been mapped for the 2016–2017 and 2017-2018 periods, respectively (from the various periods in ascending MTA) among the 240 active rock glaciers that are present in the inventory created by Bollmann *et al*.^36^ for the entire South Tyrol. We observe more events at higher elevations and a stronger correspondence of the MTA results with (potentially dangerous) active rock glaciers^37^ (Fig. 6).

#### Temporal variability

**Figure 7:**
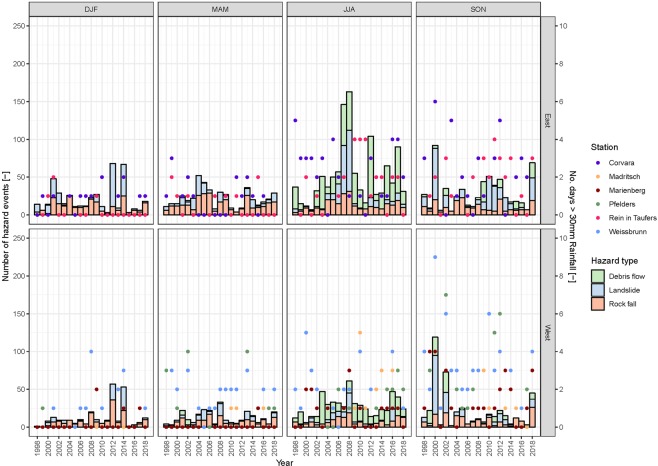
Relationship between seasonal natural hazards occurrence and rainfall.

  Figure 7 shows the temporal evolution of different event types registered in eastern and western South Tyrol between 1998 and 2018 in both event catalogues plotted together with the number of days in which 30 mm of rainfall was exceeded at the investigated meteorological stations. In terms of spatial occurrence, the eastern part of South Tyrol (i.e. the catchments of Eisack/Talfer and Rienz, see Fig. 1) features a higher number of events than the western part (Etsch-Passer catchments). It can be seen that the overall occurrence of the analysed phenomena is generally higher both in the East and the West in summer (June-July-August) and autumn (September-October-November) compared to winter (December-January-February) and spring (March-April-May). A total of 1,724 and 1,082 events were counted in summer and autumn compared with 707 and 736 entries in winter and spring, respectively. Especially the eastern part of South Tyrol appears to be prone to mass movements - mainly debris flows and landslides - during the summer season. For instance, during the summer months since the beginning of recording, 833 debris flow and landslide events were counted in the east, but only 408 in the west. In winter and spring in contrast, debris flows are nearly absent. Although the seasonal trend of landslide occurrence is less distinct, summer and autumn show higher occurrences than winter and spring.

Concerning the yearly evolution of hazard events since 1930 (see relative figure in the Supplementary Material), the increase in the number of recorded events from 1998 is mainly attributable to the rise in the information availability. However, no significant trend (according to the Mann-Kendall test) was found from 1998 to 2018 for any hazard type, in either the western or in the eastern part of South Tyrol. While an increasing trend (+1.2 event/year) in debris flow occurrence can be observed, it is associated to a relatively low (0.1) level of significance. The year with the highest total number of counted events is 2008. The years with an exceptionally high occurrence of debris flows are distributed equally over the investigated time series (i.e. in 2003, 2009, 2012 and 2017, 90 or more events were recorded). The years with the highest recordings of landslides are 2000 (173 events), 2008 (153 events), 2012 (135 events) and 2014 (102 events). However, as with the seasonal assessment, no annual trend was observed for rockfall events.

### Changing climate relationships with natural hazards

**Figure 8:**
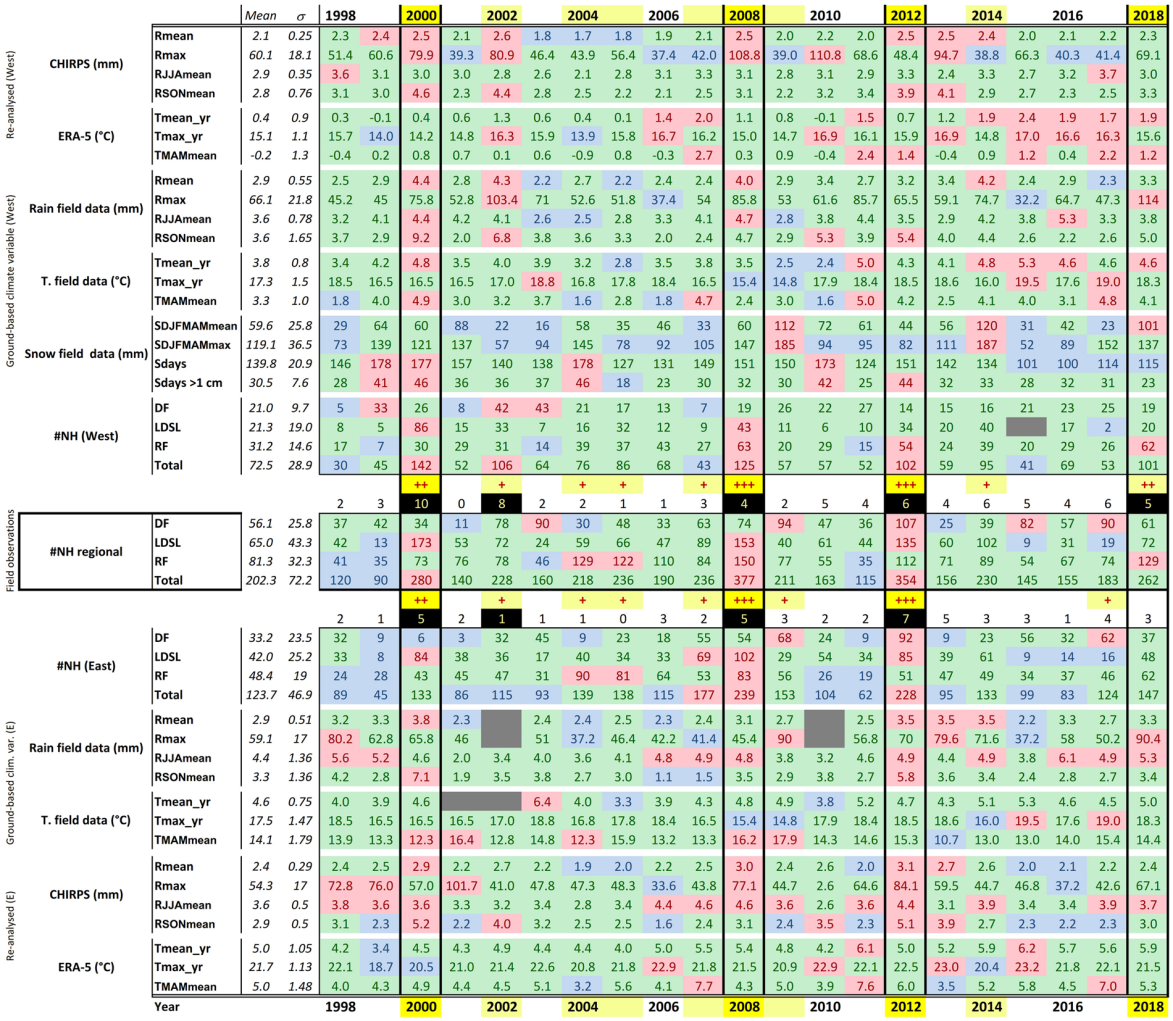
Comparison of changing climate variables at Weissbrunn (Western South Tyrol) and Corvara (Eastern South Tyrol) stations in relation to the occurrence of natural hazard types (according to the IFFI and ED30 catalogues). Red represents values significantly above the calculated mean, blue shows values significantly below the calculated average value while green shows values close to the mean (i.e. between the mean-$\sigma$ and the mean+$\sigma$). Yellow and positive signs indicates active (+), very active (++) and extremely active (+++) years. The number of climate variables corresponding to very and extremely active years are highlighted in yellow on a black background.

  Figure 8 indicates more (red), normal (green) and less (blue) active years in terms of particular NH occurrence and changes in different climate variables (or not) over time adjusted for their the calculated temporal average. Higher occurrence of NH was observed in 2000, 2008, 2012 (with more prevalence in the eastern region) and 2018 (with more evidence in the western region). While the full time series (since 1980) are considered in the calculation of the yearly mean and standard deviation, Fig. 8 focuses on the subset of the records between 1998 (since the recording of events is done in a standardized manner) and 2018. This analysis demonstrates the correspondence between abnormal yearly and seasonal meteorology (recorded in some climate variables) with peaks of NH occurrence, especially for the western region. The eastern side is not clear because of recurrent gaps in data. Generally, during active years, precipitation anomalies are mostly observed. However, seasonal peaks of rainfall are not always correlated to higher hazard occurrence at sub-regional scale (e.g. while autumn 2010 was particularly wet at Weissbrunn it is considered as normal in terms of NH occurrence).

For temperature, some correspondence between high NH occurrence and weather anomalies are observed during years with temperatures significantly above the standard mean value. These high temperature years are more frequently observed in the last 15 years. Figure 3 represents the below and above mean values for each climate parameter (both field and satellite-based) observed at Weissbrunn, which is compared with the (sub-)regional records of slope instability types in Fig. 8. While similar field and satellite-based data do not always match in terms of sign (i.e. below or above mean values; Fig. 8), some similar patterns are observed when a higher number of NH events was recorded by the authorities (see positive symbols). Indeed, during the active NH years 2000, 2002, 2012 and 2018, between 4 to 10 variables with significantly positive values (above mean+$\sigma$ value) were observed. However, this is not always the case, for example a low number of records of high climate anomalies (especially from rainfall and temperature) was observed in 2008 when 377 NH events were recorded across the whole South Tyrol province.

## Discussion and conclusion

All the events analyzed by Huggel *et al*.^7^ were characterised by warm temperatures, far above freezing level, before failure, except for the 2007 Monte Rosa landslide (Northwest Italy). In South Tyrol, 2007 was considered as an active year in terms of slope process occurrence when temperatures in spring 2007 were extraordinarily warm in central Europe. While twentieth-century warming and thermal perturbations of the ground surface, also observed in our data (especially since 2003), have penetrated to depths of a few tens of metres in high rock slopes^38^, short warm extremes have one or more years delayed effect in terms of response at depth^7^.

The World Meteorological Organization (WMO) typically recommends a minimum density of one rain gauge per 250 $k{m}^{2}$ in mountainous regions^39^. Re-analysis of mountain precipitation fields requires both interpolation between sparsely distributed gauges and extrapolation by elevation^40^. In this study, six long time series of rain gauges located in a mountainous setting were available. Different records of in situ vs. satellite-based precipitation were observed and while they had similar exceedence probability curves there was an underestimation of low magnitude events observed with CHIRPS satellite rainfall. This is considered to be because the satellite resolution smoothes local variability recorded in situ. Satellite temperature data did not always underestimate low magnitude events especially at elevations higher than 1800 m a.s.l. However, the limited optical cloud free images in mountainous areas and the difficulty in separating cloud from snow influence the accuracy of snow day records from MODIS data. Potentially better interpolation and cloud masks would enable analysis of the fraction of snow cover spatial variability and its relation to temperature anomalies.

A common issue that arises when working with NH inventory data is the incompleteness of the catalogues^41,42^. A visual comparison of the Figs. 1 and 4 reveals that events are mainly recorded near the settlement areas of South Tyrol. Therefore, it is not surprising that Fig. 5 shows a consistent decrease of database recordings with increasing altitude. This bias may be due to several factors. Firstly, events in IFFI are recorded by the South Tyrolean Geological Survey upon request of the Civil Protection Agency, whose responsibility is to protect settlement areas. Consequently, a substantial number of events are recorded only if they are causing actual or potential losses. Secondly, peripheral municipalities and their inhabitants can be considered to be more resilient towards NH since they have learned to cope with them^43^. This implies that events occurring in these areas often are not reported to the regional authorities. This bias may affect conclusions in respect of change in NH with climate, but this may be addressed by both incorporating satellite observations and using local knowledge. The increase of IFFI entries with increasing slope angles can be attributed to rock fall occurrences. Here, also local geological conditions play a role. The concentration of rock fall events in the area around the regional capital Bozen (see Fig. 1) can be explained by the presence of steep, highly fractured porphyry rock walls, susceptible to detachment of boulders. The event density (i.e. events/km^2^) reflects this with porphyry layers being the highest (i.e. 1.38) followed by plutonite rocks (0.95), calcschists (i.e. 0.57), sedimentary rocks (i.e. 0.56) and crystalline basement (i.e. 0.53).

Numerous high elevation hot-spot areas have been detected in recent years using SAR data, although MTA does not allow the recognition between different NH events (e.g. rock glaciers vs. deep-seated landslides). The incorporation of Sentinel-2 optical images to differentiate shallow landslides and to update the existing catalogue was limited by satellite imagery resolution vs event size and because rapid vegetation growth masked changes between the few available cloud free images.

Figure 7 confirms that water-driven processes leading to debris flows and (shallow) landslides exhibit a seasonal trend. Rock fall - a hazard which is strongly temperature-related - in contrast, show a less cyclical distribution. Therefore, a comparison of point-wise precipitation data with a regionally-scaled inventory on hazard occurrences requires delineation of event type and climate variable. Even so the relationship is not always consistent. The year 2008 for instance, in which the most events of the considered time period were recorded, does not show a particularly high number of days with 30 mm or more of precipitation even though the SPI-12 shows a wet month in July 2008 while SPI-3, the three month equivalent, results show very wet (at Weissbrunn) to extremely wet (at Marienberg) records for the same month.

In conclusion, there is clear evidence of rising of mean and maximum temperatures in South Tyrol, especially since 2014 and in the spring season. While snow coverage duration has decreased since 2011, snow height trend is not clear. Rainfall trends are only visible in terms of increase of average duration of events for two stations (i.e. Pfelders and Weissbrunn) while no trend is visible at Marienberg. If the magnitude of events is analysed an increasing trend is visible only at one station (Weissbrunn). Some abnormally wet years are recorded, especially in 2001, 2009 and 2014 (considering SPI-12) but the pattern is very variable. In relation to natural hazard occurrence, it is difficult to observe an unequivocal association with changes in climate, although in some cases there are indications that NH do respond to meteorological change that is also visible in climate records. Thus, the monitoring of regional-scale climate change effects on slope instabilities is challenging, even in one of the most studied area of the world. This is considered mainly due to the spatial variability of rain and snow precipitation as well as NH catalogue completeness. This is compounded by the availability of long time series of meteorological observations above 1000 m a.s.l. where we expect more natural hazard phenomena to occur in response to changing climate. With regard to NH inventories the satellite (SAR) data provide complementary information and are particularly useful for sparsely populated, higher altitudes which are not monitored well in inventories. This is particularly the case for rock glaciers and slow moving events, although new methods are needed to delineate natural hazard type. Further analysis and longer temporal windows of SAR data for MTA will be useful to better assess the evolution of these NH events at higher elevations.

## Methods

**Figure 9:**
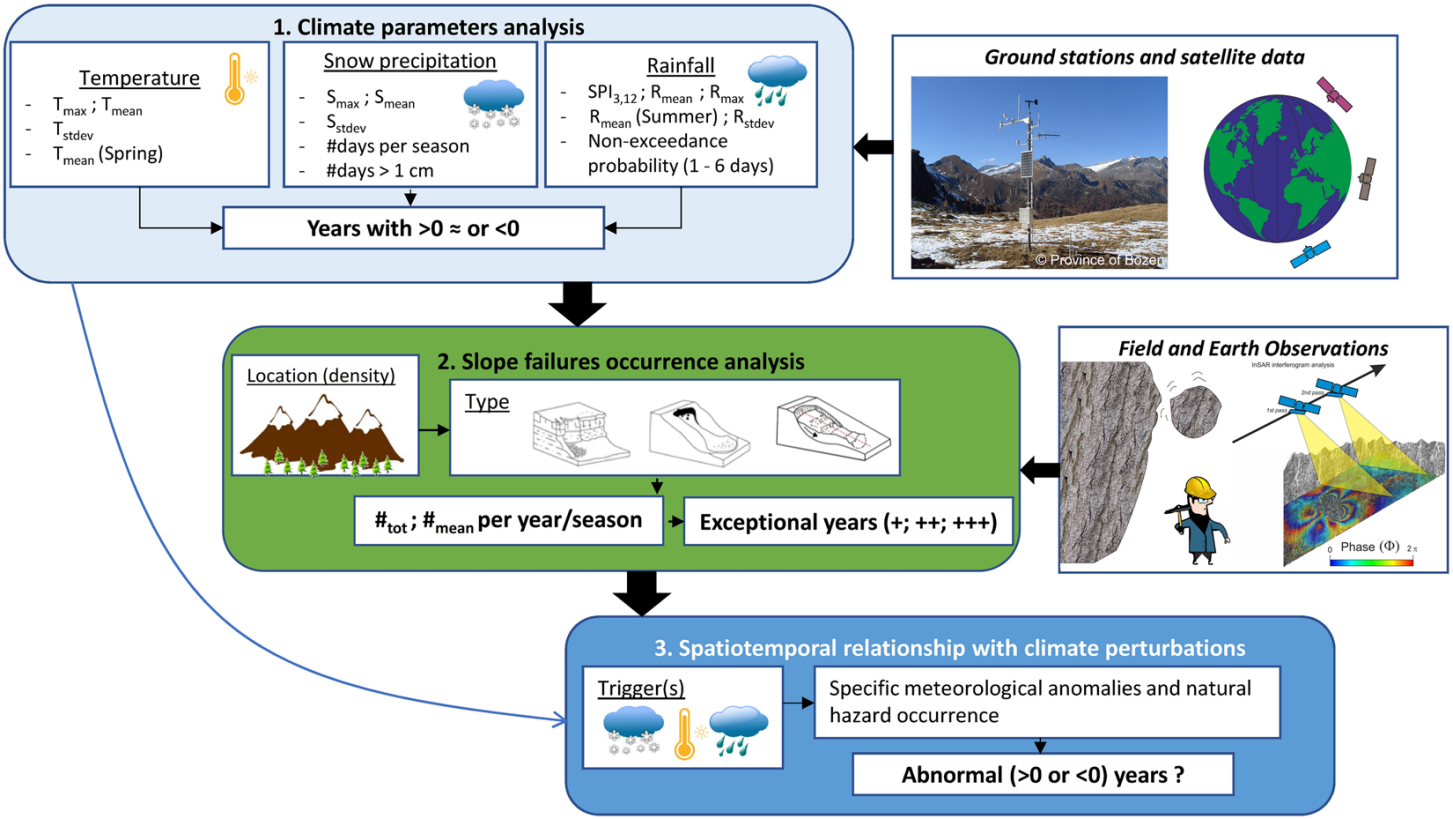
Working flowchart to interpret data and results investigating climate change effect on natural hazard occurrence. Weather station image courtesy of R.Nadelet (Autonomous Province of Bozen).

Figure 9 introduces the workflow representing the three main steps of data analysis leading to the result interpretation above. A description of the applied data sets (satellite, reanalysis and field data) can be found in tabular form in the Supplementary Material.

### Rainfall return period using the Gumbel distribution

Short-term climatic trends may be observed through the evaluation of the return period of extreme events on several decades of rainfall data. Fisher and Tippett^44^ developed frequency distribution analysis of maximum values, popularized by Gumbel^45,46^ in the fields of hydrology and meteorology. Gumbel’s distribution is an approximation of the distribution of the maxima of a sample of independent random variables^47^. The Gumbel’s distribution is asymmetric and extends towards the high values on the abscissa. The calculation of return periods using Gumbel’s law needs a series of continuous meteorological data over a sufficiently long period of daily values. The moments method was used to estimate the return period of rainfall daily exceedence threshold values with the methodology described by Vivekanandan^48^, on both ground and satellite daily rainfall series. The annual maximum series model was implemented from ranked values in order to estimate exceedance probabilities for each dataset. Comparison between ground-based observations and satellite-based data was then possible, restricting data ranges to the same temporal period.

### Trend analysis of rainfall records

In order to analyse possible variations in rainfall conditions in the study area, single rainfall events, in terms of their duration and cumulative rainfall, were reconstructed from continuous rainfall series recorded at the Marienberg, Pfelders, and Weissbrunn stations. The length of the rainfall series are different for the three stations: data are available since in 1921, 1924 and 1961 in Pfelders, Marienberg and Weissbrunn, respectively. All series end in December 2018. For the reconstruction of rainfall events, the tool proposed by Melillo *et al*.^49^ was used. The tool receives as input continuous rainfall series and reconstructs distinct rainfall events, calculating their duration (*D*, in h, or in days) and cumulative event rainfall (*E*, in mm), using parameters to account for different seasonal and climatic settings of the study area. A rainfall event is defined as a period of continuous rainfall separated from the preceding and the successive events by dry (no-rain) periods. The length of the dry period depends on the seasonal and climatic conditions of the investigated area. Specifically, the length of the two seasonal periods (dry and wet) was selected in accordance with Peruccacci *et al*.^50^: given that the study area is located in northern Italy, the dry period was set from June to September and the wet period from October to May. Moreover, a period of two (four) days without rainfall was set for the dry (wet) season. Finally, to determine the possible existence of temporal trends in climate variables, the time series were analysed using least squares to calculate slopes, and their statistical significance assessed with the Mann-Kendall non-parametric test^34,35^. This test is applicable when the values of a time series can be assumed to obey to a continuous monotonic increasing or decreasing function of time, with residuals assumed to be from the same distribution with zero mean and variance constant in time. Moreover, it does not require that the data be normally distributed or linear, it can be used also with time series including missing data, and it guarantees robustness even in the presence of outliers. The test consists in testing the null hypothesis of no trend, i.e. the observations are randomly ordered in time. The statistical significance level defines the probability that the values are from a random distribution. The lower the value is, the higher the significance of the trend.

### Single climate parameter anomalies

The SPI is simply the difference of precipitation from the mean for a specified time period divided by the standard deviation where the mean and standard deviation are determined from past records^51^. SPI is the result of the normal quantile transformation applied to a fitted parametric distribution of the original value^52^. The main advantage of SPI exists in a quantitative analysis of shortage (or exceedence) of precipitation with reference to a climatic mean state (e.g. annual precipitation itself dominated by summer precipitation). SPI were calculated for the different stations according to CHIRPS and in situ data for 3-month and 12-month periods using https://drought.unl.edu/droughtmonitoring/SPI/SPIProgram.aspx. In terms of NH assessment, SPIs for short accumulation periods (e.g. SPI-3) are indicators for immediate impacts such as reduced soil moisture, snowpack and flow in smaller streams that could trigger shallow slope instabilities for instance. SPIs for long accumulation periods (e.g. SPI-12) are indicators for reduced reservoir and groundwater recharge, and possible slow slope deformation such as deep-seated (or rock glacier) ground surface deformation, for example.

The single variables (e.g. rainfall, temperature and snow fall) were analysed following Huang^53^. Each variable was converted to standardised normal variable following the Eq. (1)

1 $$x=\left(\frac{X-\mu }{\sigma }\right)$$

where $X$ is the standardised value of $X$, the climatic annual variable, $\mu$ is the expectation of the distribution and $\sigma$ is its standard deviation.

### Analysis of natural hazard event databases

The regional authorities of South Tyrol compile two event databases: IFFI and ED30. Due to differences in time range, recorded hazard types and hazard designations as well as duplicate events appearing in both catalogues, a workflow to remove these inconsistencies was developed. In a pre-processing step, both catalogues were reduced to three event types, namely debris flows, landslides and rock falls. Other types of hazards such as floods or snow avalanches were excluded. A duplicate event of one catalogue was labelled as such if it had a matching counterpart in terms of event type, date and location in the other catalogue. The identified duplicate events were removed from one catalogue. We also calculated an event density for the five geological units (in events/km^2^) crystalline basement, porphyry, sedimentary, plutonite and calcschists available from the South Tyrol province authority. A detailed description of the work flow to unify both catalogues can be found in the additional material. A heatmap renderer from QGIS was used to create density maps of the NH catalogues (i.e. landslides, debris flows, rock falls) and rock glaciers from a separate inventory.

The seasonal and yearly evolution of debris flows, landslides and rock fall were plotted against the number of days, in which more than 30 mm of rainfall was recorded (Fig. 7). We selected this threshold heuristically, supported by the work of Peruccacci *et al*.^50^, which suggests a value of about 30 mm of cumulative rainfall in one day as a threshold for the initiation of landslides in Italian Alpine areas.

### Earth Observation for hazard detection

MTA has been used to detect hotspot areas, update an inventory^54^ and determine the completeness of the available catalogues. De Luca *et al*.^55^ provides an overview of the G-POD environment (a former version of the Geohazard Exploitation Platform (GEP)) describing in detail the features and characteristics of a Parallel Small BAseline Subset (P-SBAS) InSAR web tool and its implementation. In this work the FASTVEL algorithm^24^ which has been developed by TRE-Altamira for generating differential interferograms or Persistant Scatterrer Interferometry (PSI)-based mean displacement velocity maps from a set of Sentinel-1 or ASAR images is used within the GEP (https://geohazards-tep.eu/). The MTA mode produces the following output results: (1) a ground displacement velocity map, (2) the updated topography, including a reference DEM with height uncertainty, (3) a CSV file with the main information of PSI products in the LOS (Line Of Sight). The input parameters include the selection of the area of interest (see Figs. 4 and 6, the reference point coordinates (e.g. latitude: 46.627${}^{\circ }$N, longitude: 10.775${}^{\circ }$E), the maximal temporal baseline (here 200 days), the maximal perpendicular baseline (here 400 m), the coherence threshold (0.45) and the Atmospheric Phase Screen Correlation distance (2000).

MTA with the FASTVEL algorithm enables a fast detection of hot-spot areas corresponding to slow moving landslides (or active rock glaciers) affecting South Tyrol province (Fig. 6). Each area comprising at least three pixels (i.e. 4,800 m^2^) and recording more than 2 cm of displacement per year was mapped to the topographic properties (using the DEM) in the existing inventories. A spatial and temporal subset of the event databases IFFI and ED30 was created in order to provide a comparison with remotely detected slope movements (Fig. 5). Criteria of presence/absence of NH events, rock glaciers and MTA-based hot-spots areas (considering other periods and track) were mentioned in the attribute table of the specific MTA-based shape files presented in Figure 6B,D.

## Supplementary information

Supplementary Information.

## Data Availability

Contains Copernicus Sentinel-1 data 2015–2017, freely provided by ESA and processed in GEP https://geohazards-tep.eu. The natural hazard event catalogues as well as temperature, rain and snow precipitation data were provided upon request by the responsible regional authorities (see acknowledgements). The regional digital elevation model, lithological layers and the rock glacier inventory of South Tyrol were downloaded from the South Tyrolean public administration (http://geokatalog.buergernetz.bz.it/geokatalog/). CHIRPS (daily v2.0 data with improved temporal downscaling procedure^56^) were collected on http://iridl.ldeo.columbia.edu/SOURCES/.UCSB/.CHIRPS/.v2p0/.daily-improved/.global/.0p05/.prcp/. ERA5 and TERRA-AQUA MODIS data were extracted from stations’ benchmarks using the ADAM platform (https://adamplatform.eu). Snow products were downloaded from the CryoLand (http://neso1.cryoland.enveo.at/cryoclient/) and Eurac Research Sentinel Alpine Observatory (SAO) servers (http://saocompute.eurac.edu/rasdaman/ows).
