# Author Correction: Changes in climate patterns and their association to natural hazard distribution in South Tyrol (Eastern Italian Alps)

**DOI:** 10.1038/s41598-020-66842-9

**Published:** 2020-06-23

**Authors:** Romy Schlögel, Christian Kofler, Stefano Luigi Gariano, Jean Van Campenhout, Stephen Plummer

**Affiliations:** 1 ESA Climate Office, ECSAT, Didcot-Harwell, OX11 0FD United Kingdom; 2 Institute for Earth Observation, Eurac Research, Bozen, 39100 Italy; 3 CNR IRPI, Perugia, 06127 Italy; 4 Department of Geography, University of Liege, Liege, 4000 Belgium; 5 Faculty of Science and Technology, Free University of Bozen-Bolzano, Bozen, 39100 Italy; 6 Present Address: Division for Satellite Analysis and Applied Research (UNOSAT), UNITAR, United Nations Office at Nairobi, Nairobi, 00200 Kenya; 7 Data Applications Division, ESRIN, Frascati, 00044 Italy

Correction to: *Scientific Reports* 10.1038/s41598-020-61615-w, published online 19 March 2020

The Acknowledgements section in this Article contains errors.

“The authors acknowledge the Office for Geology and Building Materials Testing (D. Costantini), the Regional Warning Centre (O. Formaggioni) and the Hydrographic Office (R. Nadalet) of the Autonomous Province of Bolzano/Bozen - South Tyrol for providing the event catalogues, the meteorological data and useful comments on the manuscript. We acknowledge F. Pacini and H. Gaumont (Terradue) for developing and maintaining the GEP as well as P. Blanco (Tre-Altamira) in FASTVEL algorithm use. We thank M. Melillo (CNR IRPI) for helping in the reconstruction of rainfall events. We acknowledge S. Natali (Sistema) for enabling climate data access on ADAM platform and A. Jacob (Eurac Research) for the collection and interpretation of updated snow cover data. R.S.’s work was supported by an ESA Climate Office research fellowship. S.L.G. was granted by a Regione Puglia - Civil Protection Department research fellow (project id: B82F16003840006). C.K. was supported by the Stiftung Südtiroler Sparkasse/Fondazione Cassa di Risparmio di Bolzano PhD funding program on future-relevant topics for South Tyrol (grant number: 2017.0160).”

should read:

“The authors acknowledge the Office for Geology and Building Materials Testing (D. Costantini), the Regional Warning Centre (O. Formaggioni) and the Hydrographic Office (R. Nadalet) of the Autonomous Province of Bolzano/Bozen - South Tyrol for providing the event catalogues, the meteorological data and useful comments on the manuscript. We acknowledge F. Pacini and H. Caumont (Terradue) for developing and maintaining the GEP as well as P. Blanco (Tre-Altamira) in FASTVEL algorithm use. We thank M. Melillo (CNR IRPI) for helping in the reconstruction of rainfall events. We acknowledge S. Natali (Sistema) for enabling climate data access on ADAM platform and A. Jacob (Eurac Research) for the collection and interpretation of updated snow cover data. R.S.’s work was supported by an ESA Climate Office research fellowship. S.L.G. was granted by a Regione Puglia - Civil Protection Department research fellow (project id: B82F16003840006). C.K. was supported by the Stiftung Südtiroler Sparkasse/Fondazione Cassa di Risparmio di Bolzano PhD funding program on future-relevant topics for South Tyrol (grant number: 2017.0160).”

