# Supplementary material for: Changes in climate patterns and their association to natural hazard distribution in South Tyrol (Eastern Italian Alps)
Source: Sci Rep. 2020 Mar 19;10:5022. doi: 10.1038/s41598-020-61615-w (PMC7081291; doi:10.1038/s41598-020-61615-w)
Supplement: Supplementary file 1 — Supplementary Information. [file 41598_2020_61615_MOESM1_ESM.pdf]

Changes in climate patterns and their association to natural hazard distribution in South Tyrol (Eastern Italian Alps)

Romy Schlögel[1,*], Christian Kofler[2,5], Stefano Luigi Gariano[3], Jean Van Campenhout[4], Stephen Plummer[1]

[1]ESA Climate Office, ECSAT, Didcot-Harwell, OX11 0FD, United Kingdom
[2]Institute for Earth Observation, Eurac Research, Bozen, 39100, Italy
[3]CNR IRPI, Perugia, 06127, Italy
[4]Department of Geography, University of Liege, Liege, 4000, Belgium
[5]Faculty of Science and Technology, Free University of Bozen-Bolzano, Bozen, 39100, Italy

[*]Corresponding author: romy.schlogel@gmail.com

Supplementary material

Climate data

The climate-related data used in the frame of this analysis are presented in Table 1. Daily CHIRPS v2.0 data with an improved temporal downscaling procedure[1] measures InfraRed precipitation constrained by station data. These data were chosen as wet season precipitation estimates from climate models tended to have reasonably high correlations with observed data in Europe[2] and long time series. Daily ERA-5 data [1] were downloaded from the Climate Data Store [2] using APIs [3].
The Terra satellite for NASA's Earth Observation System (EOS) program, launched in 1999, aims to study the changing global land surface[3]. The Moderate resolution Imaging Spectroradiometer (MODIS) collects data for 36 spectral bands at various resolution viewing the Earth with a daily return-period while we extracted daily snow products at snow stations (i.e. Pfelders, Madritsch and Rossbaenke) from three different platforms: ADAM, CryoLand and Eurac research (see links in *Data availability* section). The NASA products [4] consider both Terra and Aqua satellites to assign "snow" and "no snow" values, identified using the Normalized Difference Snow Index (NDSI) with consideration of the overall quality of algorithm result. This snow cover dataset contains a lot of data gaps due to the cloud coverage of the area and issues of differentiation between cloud and snow. While the CryoLand snow products are now available at the Alps-scale with a 250-m resolution since 2010 [5], we used the homogenized Fractional Snow Cover (FSC) product over the Pan-European area at 500 m available since 2000 [6]. We extracted values from each of the stations and interpolated number of snow days (including cloud cover records) having a FSC of more than 50 percent for each winter season (usually from September of year-1 to June of the considered year). This interpolation might result in overestimating the Sdays while snow often melts between the first snow (in autumn) and the start of the snow season and late snow fall might happen in late spring.
Eurac Research (Bolzano) produces a real-time Snow Cover Area (SCA) daily product at a fine 250-m resolution[4]. Only SCA labelled as "snow" containing quality flags (excluding worst snow quality and satellite viewing geometry) were considered. Indeed, cloud days (e.g. between 164 to 236 days per year at Pfelders station) over one year generally make the interpolation difficult. While datasets comparison show differences due to the interpolation of the CryoLand dataset (see Fig. 2), a good general correspondence is found (e.g. at Pfelders on 11/01/2003: 33 mm, 0.675, 100 percent, snow presence with 243/255 Quality of Snow Cover Estimation flag considering ground measurement, NSDI, FSC and SCA values, respectively). From these raw data, additional experiments are required in order to enable the investigation of spatiotemporal variations of snow cover in relation to increases in temperature.

Table 1. Remote climate datasets and properties.

Climate data	Type/Sensor	Period coverage	Frequency	Resolution
Precipitation	CHIRPS	1981-2018	Daily	0.05° x 0.05°
Temperature	ERA-5	1980-2018	Daily	0.25° x 0.25°
NDSI (Nasa)	TERRA-AQUA MODIS	2001-2018	Daily	0.05° x 0.05°
FSC (CryoLand)	MODIS	2001-2018	Daily	0.05° x 0.05°
SCA (Eurac)	MODIS	2003-2018	Daily	0.025° x 0.025°

In the South Tyrol province, 35 measurement stations are currently monitoring weather parameters in mountainous areas [7]. Table 2 presents the different ground stations considered in this study, their coverage period and location. Four meteorological stations (located in Western South Tyrol) have long term rainfall and temperature monitoring records: Madritsch, Pfelders, Rossbaenke and Weissbrunn and two meteorological stations (located in Eastern South Tyrol) have been considered: Corvara (1558 m a.s.l.) and Rein in Taufers (1562 m a.s.l.).Three high altitude snow precipitation gauges are available, covering the period since December 1981 (Pfelders; 1623 m a.s.l.), 1996 (Madritsch; 2825 m a.s.l.) and 2001 (Rossbaenke; 2255 m a.s.l.) onwards. While Weissbruun (1876 m a.s.l.) station is located 2 km away from Rossbaenke station, we consider that the amount of snow and rainfall are similar in both locations.

While the main paper shows only results of parameters measured at Weissbrunn station, the temporal evolution of climate variables (i.e. yearly standard normal) measured at other stations is presented in Figures 1 and 2.

[1] https://cds.climate.copernicus.eu/cdsapp#!/dataset/reanalysis-era5-single-levels?tab=overview
[2] https://cds.climate.copernicus.eu/#!/home
[3] https://cds.climate.copernicus.eu/api-how-to
[4] https://nsidc.org/data/mod10a1, https://nsidc.org/data/myd10a1
[5] http://cryoland.enveo.at/services/snow-services/regional-snow-products/fractional-snow-cover-over-alps
[6] http://cryoland.enveo.at/services/snow-services/pan-european-snow-products/fsc-pan-europe
[7] http://weather.provinz.bz.it/mountain-wind-stations.asp

Table 2. Local weather stations and properties.

Climate data	Station	Starting measurement	Frequency	Localisation
Rain/Snow/T.	Madritsch	03/1996 (S,T), 2008 (R)	Daily	46.493 10.615
Rain/Snow/T.	Pfelders	01/1921 (R), 03/1978(T), 12/1981(S)	Daily	46.797 11.088 (R/T)
Snow/T.	Rossbaenke	11/2001	Daily	46.469 10.819
Rain/T.	Weissbrunn	01/1961 (R), 01/1983 (T)	Daily	46.487 10.832
Rain	Marienberg	01/1924 (R)	Daily	46.706 10.521
Rain/T.	Corvara	02/1981 (R), 01/1989 (T)	Daily	46.525 11.875
Rain/T.	Rein in Taufers	01/1981 (R), 11/2008 (T)	Daily	46.925 12.075

Natural hazard event databases

Two event databases on natural hazards supported this research: the "Inventario dei Fenomeni Franosi in Italia" (IFFI) and the "Ereignis Dokumentation 30" (ED30). IFFI is a nationwide project initiated by the National Italian Institute for environmental protection and research (ISPRA) and aims to establish a national database on gravitational mass movements. However, the mapping and maintenance of the IFFI database has been delegated to the regional geological surveys. The IFFI-project was launched in 1995. ED30 is a project launched in 1998 by the Regional Agency for Civil Protection of South Tyrol. Since that year, the database is systematically updated with field reports, photographs and GIS-information related to floods, debris flows, shallow landslides and snow avalanches. In addition, historical events have been reconstructed during an archive study.
While the availability two event databases certainly provided a more complete picture of the spatio-temporal occurrence of natural hazards in the region, it required also the implementation of a method that allows the homogenisation of the catalogues and the identification of events that were recorded by both managing authorities and hence appear in both catalogues.
Figure 3 shows the workflow that was implemented to homogenize the two databases and to filter identical events. Preceded by a preprocessing step, an event recorded in both databases is identified according to thematical, temporal and spatial criteria. Accordingly, we build an algorithm that:

(i) Reduces in a preprocessing step both databases to a period of systematic recordings that overlap in time. For the present case, this was the period from 01.01.1998 to 31.12.2018. IFFI is managed systematically since 1995, and thus had to be reduced to the systematic recording period of ED30, from 1998 on. In addition, the terminology related to event types had to be homogenized; while IFFI is purely focused on different types of landslides, ED30 covers a broader range of event types, including different types of hydraulic hazards and snow avalanches. This required a reduction or exclusion of hazard types in order to make the databases comparable (see Table 3). For the present study, we reduced the broad spectrum of hazard classes to three rather general event types: landslides, debris flows and rock fall. Hazard types that could not be summarized by one of these three classes such as snow avalanches or floods were labelled as "Other". This latter class was excluded from the data analysis.

(ii) Subsets of both databases according to the same event type, selected by the user (e.g. debris flows).

(iii) Searches for matching dates within the subset of the databases of the same event type. The events in IFFI that occur thematically and temporally also in ED30 were extracted from the former one and written into a new data frame.

(iv) Identifies entries of this newly created subset of IFFI that are located within a spatial buffer of 100 m around the points of ED30.

(v) Removes the entries of IFFI that occur thematically, temporally and spatially also in ED30 from the initial IFFI data base and joins it to ED30.

Table 3. Conversion table of event classes.

Database	Original designation	New designation
IFFI	Area susceptible to rock fall	Other
	Area susceptible to shallow landsliding	Other
	Area susceptible to rock fall	Other
	Area susceptible to ground subsidence	Other
	Slow moving flow	Landslide
	Fast moving flow	Debris flow
	Unchannelized debris flow	Debris flow
	Debris flow	Debris flow
	Complex landslide	Landslide
	Rock fall	Rock fall
	Rock fall/toppling	Rock fall
	Deep seated gravitational slope deformation	Landslide
	Lateral spread	Other
	Rotational/translational sliding	Landslide
	Ground subsidence	Other
ED30	Flood	Other
	Torrential flood	Other
	Urban/pluvial flood	Other
	Debris flow	Debris flow
	Slow moving flow	Landslide
	Fast moving flow	Debris flow
	Unchannelized debris flow	Debris flow
	Debris flow	Debris flow
	Rock fall	Rock fall
	Bank erosion	Other
	Landslide	Landslide
	Snow avalanche	Other
	Undefined	Other
	Other	Other

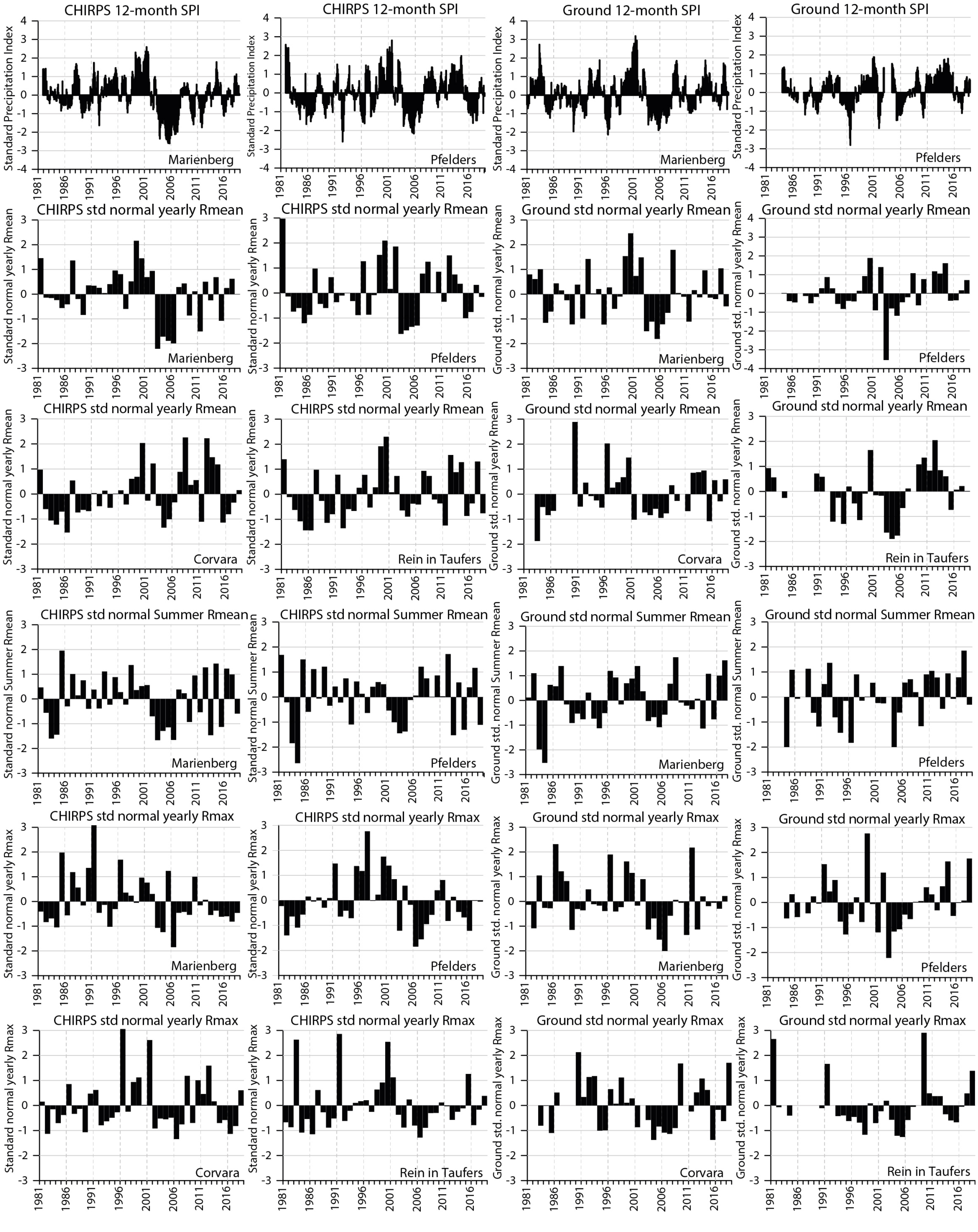

Figure 1. Climate variables evolution at Marienberg, Pfelders, Madritsch, Corvara and Rein in Taufers stations (Part 1).

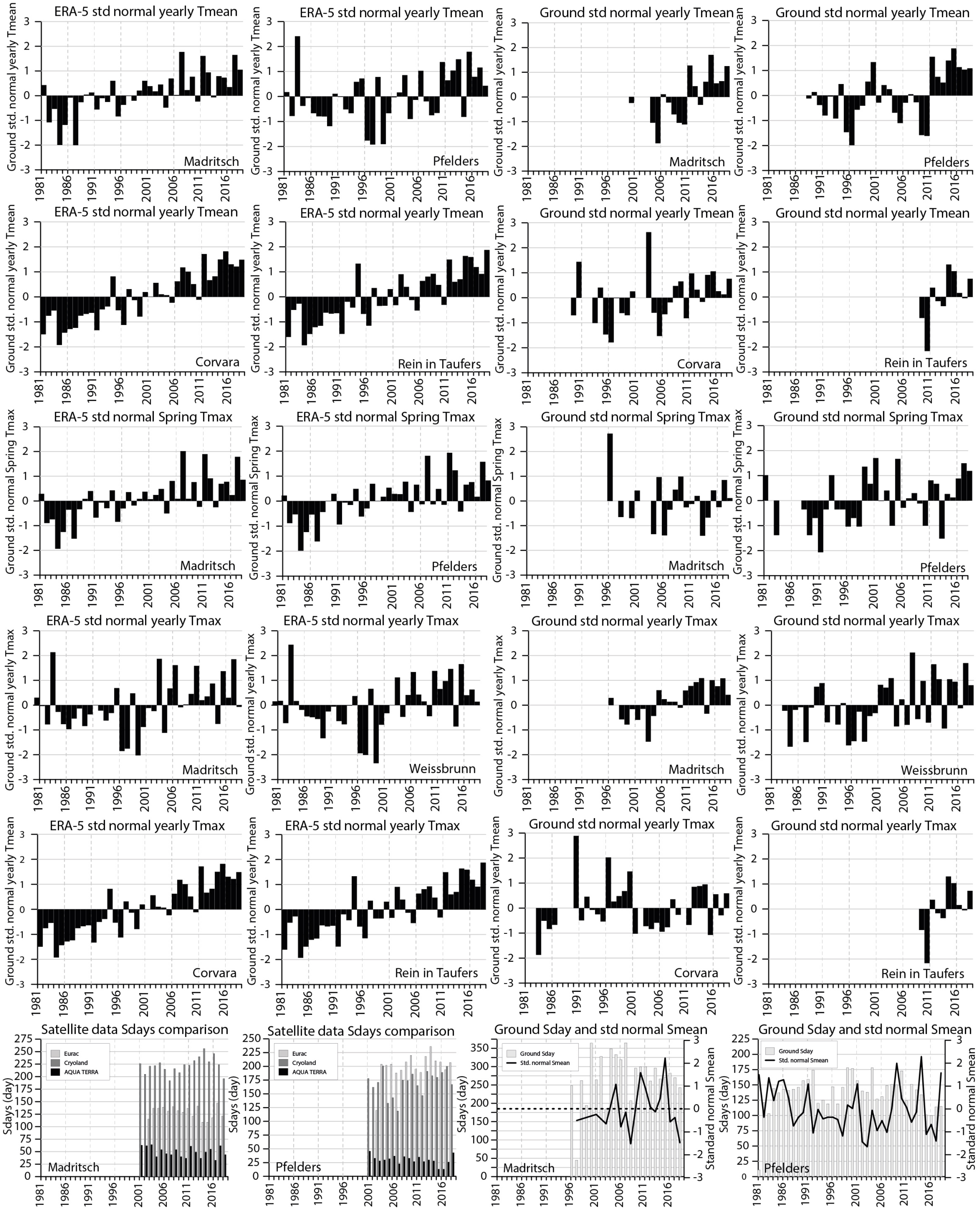

Figure 2. Climate variables evolution at Pfelders, Madritsch, Corvara, Rein in Taufers and Weissbrunn stations (Part 2).

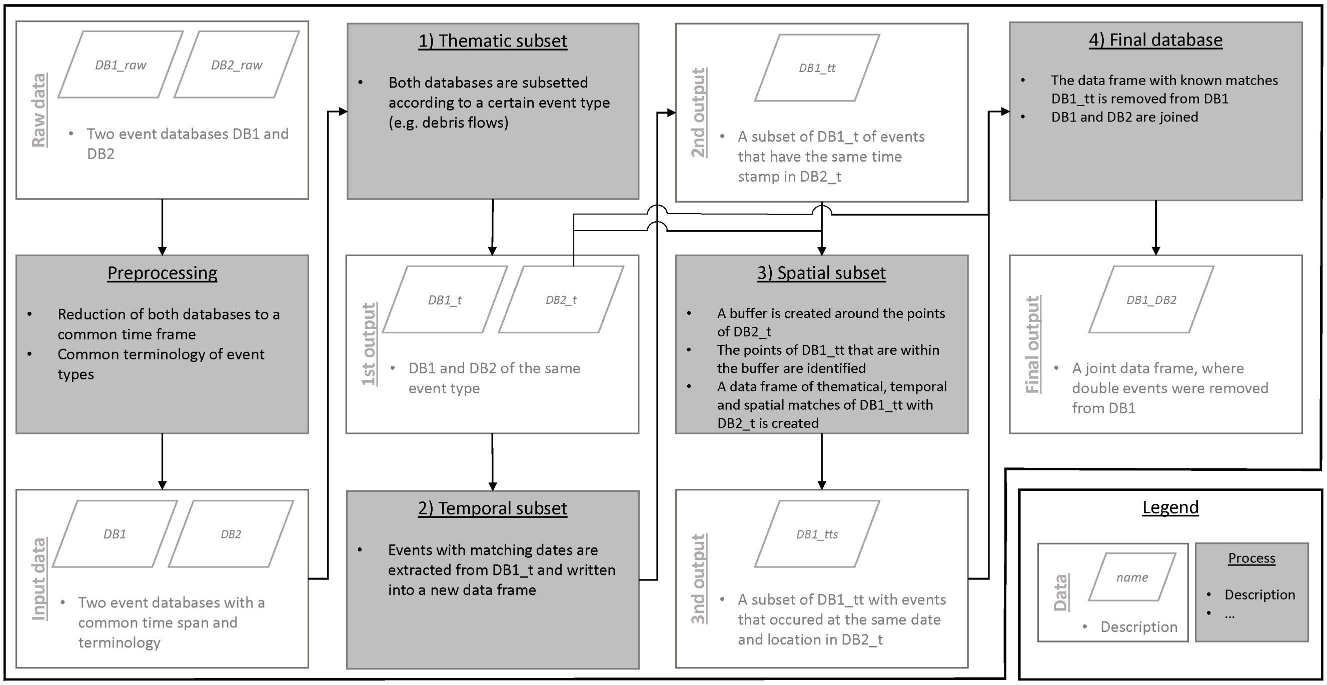

Figure 3. Applied workflow to identify double events in both databases

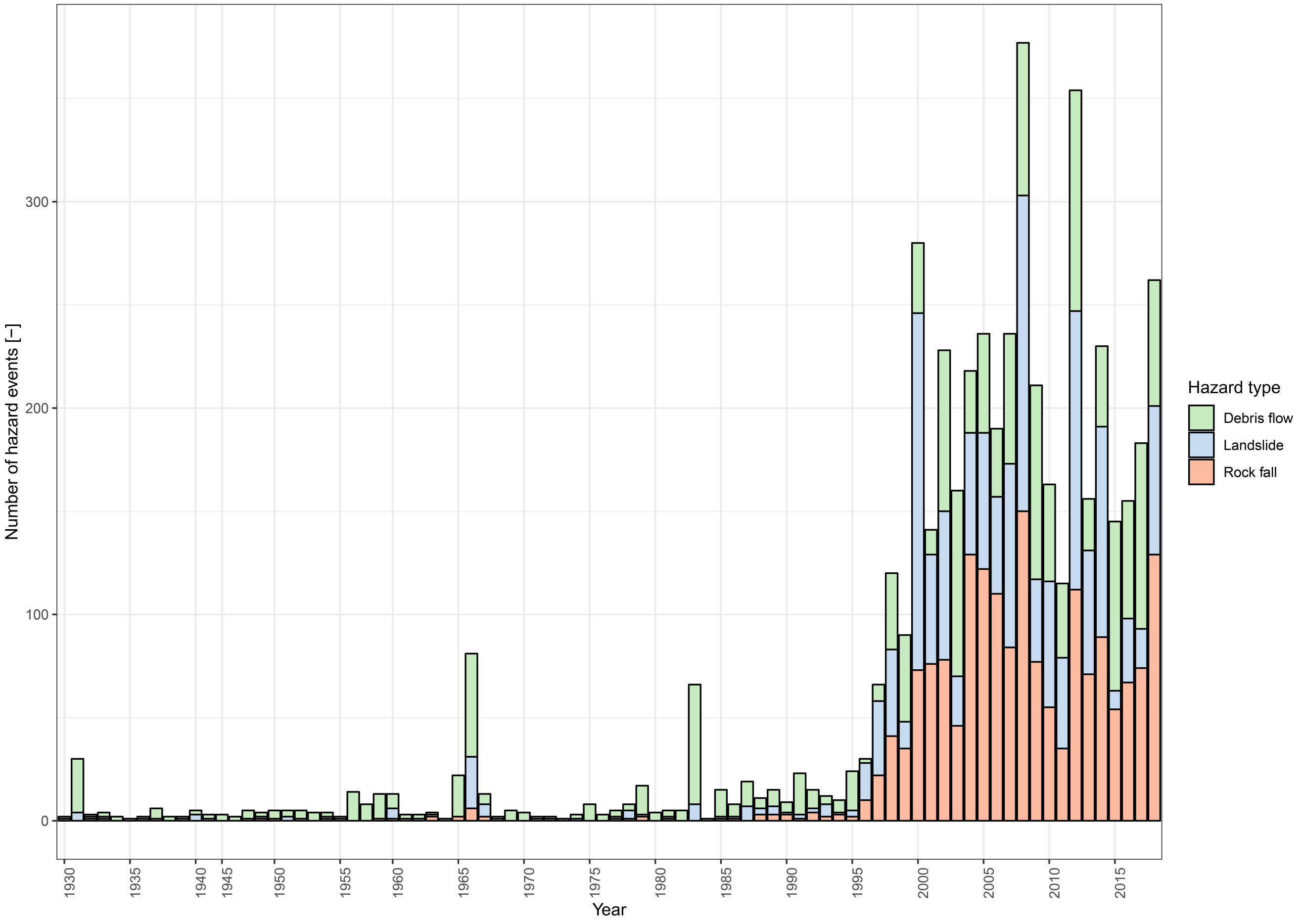

Figure 4. Time series of recorded events from 1930 onwards

References

1. Funk, C. C. *et al.* A quasi-global precipitation time series for drought monitoring: U.S. Geological Survey Data Series 832. *Usgs* 4, DOI: 10.3133/ds832 (2014).

2. Funk, C. *et al.* The climate hazards infrared precipitation with stations - A new environmental record for monitoring extremes. *Sci. Data* **2**, 1–21, DOI: 10.1038/sdata.2015.66 (2015).

3. Bishop, M. P., Colby, J. D., Luvall, J. C., Quattrochi, D. and Rickman, D. L. Remote-sensing science and technology for studying mountain environments. In *Geographic Information Science and Mountain Geomorphology*, 147–187 (Praxos Publising, Chichester, UK, 2004).

4. Thirel, G. *et al.* Assessing the quality of a real-time Snow Cover Area product for hydrological applications. *Remote. Sens. Environ.* **127**, 271–287, DOI: 10.1016/j.rse.2012.09.006 (2012).
